# The ESCRT-III Protein *Chmp1* Regulates Lipid Storage in the *Drosophila* Fat Body

**DOI:** 10.3390/medsci11010005

**Published:** 2022-12-26

**Authors:** Austin M. Fruin, Kelly E. Leon, Justin R. DiAngelo

**Affiliations:** Division of Science, Pennsylvania State University, Berks Campus, Reading, PA 19610, USA

**Keywords:** triglyceride, *Drosophila*, *Chmp1*, fat body

## Abstract

Defects in how excess nutrients are stored as triglycerides can result in several diseases including obesity, heart disease, and diabetes. Understanding the genes responsible for normal lipid homeostasis will help understand the pathogenesis of these diseases. RNAi screens performed in *Drosophila* cells identified genes involved in vesicle formation and protein sorting as important for the formation of lipid droplets; however, all of the vesicular trafficking proteins that regulate lipid storage are unknown. Here, we characterize the function of the *Drosophila* Charged multivesicular protein 1 (*Chmp1*) gene in regulating fat storage. *Chmp1* is a member of the ESCRT-III complex that targets membrane localized signaling receptors to intralumenal vesicles in the multivesicular body of the endosome and then ultimately to the lysosome for degradation. When *Chmp1* levels are decreased specifically in the fly fat body, triglyceride accumulates while fat-body-specific *Chmp1* overexpression decreases triglycerides. *Chmp1* controls triglyceride storage by regulating the number and size of fat body cells produced and not by altering food consumption or lipid metabolic enzyme gene expression. Together, these data uncover a novel function for *Chmp1* in controlling lipid storage in *Drosophila* and supports the role of the endomembrane system in regulating metabolic homeostasis.

## 1. Introduction

The ability of an organism to regulate its consumption and storage of nutrients is essential for long-term survival when access to food is sporadic. However, in conditions where food is abundant, like in current Western society, excess nutrients are stored as triglycerides resulting in obesity [1]. Obesity predisposes humans to heart disease, type II diabetes and cancer, some of the leading causes of death in the United States [2]. Understanding the mechanisms of how lipid homeostasis is regulated may allow for the identification of potential therapeutic targets for this deadly disease.

The fruit fly, *Drosophila melanogaster*, is used as a model system to study lipid metabolism due to its similarity to humans in terms of genes and metabolic biology [3,4]. The *Drosophila* system has been used to perform genetic screens to identify genes important for the storage of lipids in both cultured cells and intact flies [5,6,7]. From these screens, a group of genes involved in forming the vesicular coat protein complex COPI have been implicated in the regulation of lipid storage [5,6]. Proteins that are destined to be secreted from eukaryotic cells move throughout the endomembrane system starting by being translated into the ER, then traveling to the Golgi apparatus and ultimately being secreted via exocytosis [8]. These proteins travel throughout the endomembrane system in vesicles that are formed from and targeted to different cellular compartments using many classes of proteins such as coat proteins, Rabs and SNAREs [8]. While members of the COPI coat protein complex have been shown to regulate lipid droplet formation [5,6], whether other proteins involved in the movement of cargos throughout the endomembrane system regulate lipid storage is not well understood.

Many membrane-localized signaling receptors are taken into cells through endocytosis and are recycled back to the plasma membrane when necessary. However, some signaling receptors are monoubiquitinated targeting them for endocytosis and degradation in the lysosome [9]. These monoubiquitinated receptors are localized to intralumenal vesicles (ILVs) of the endosomal compartment called the multivesicular body (MVB) [10]. Four different endosomal sorting complex required for transport (ESCRT) complexes (ESCRT-0, -I, -II, -III) are required for the localization of these signaling receptors to ILVs [9]. ESCRT-0 complex proteins function to cluster the monoubiquitinated receptors, while ESCRT-I and ESCRT-II complex proteins work together to bud the endosomal compartment membrane. ESCRT-III complex proteins then function to regulate membrane scission at a bud neck and promote ESCRT complex disassembly ultimately forming an endocytic vesicle [9]. In addition to endosomal sorting, ESCRT-III proteins are involved in a number of biological processes such as cytokinesis, neuronal pruning, nuclear envelope repair and maintenance, autophagy, viral budding and replication, and membrane shaping [11]. ESCRT proteins have also been identified as being involved in lipid droplet utilization in yeast [12]; however, whether these proteins function to regulate lipid homeostasis in a multicellular metazoan is not known.

In this study, we characterize the role of Charged multivesicular body protein 1 (*Chmp1*) in regulating lipid storage in *Drosophila*. *Chmp1* is a member of the ESCRT-III complex and functions in ESCRT complex protein dissociation by binding to and recruiting the ATPase, Vps4 [13,14]. Decreasing *Chmp1* levels specifically in the fat body using RNAi results in triglyceride accumulation, while fat-body-specific *Chmp1* overexpression results in a lean phenotype. *Chmp1* does not regulate lipid storage by affecting food consumption or the expression of lipid metabolic enzyme genes; however, fat cell size and number are altered in *Chmp1-RNAi* and *Chmp1* overexpressing flies, providing a potential mechanism for the triglyceride phenotypes observed in these flies. These results uncover a novel role for the ESCRT-III complex protein, *Chmp1*, in regulating lipid homeostasis in the fly fat body.

## 2. Materials and Methods

### 2.1. Fly Genetics

The following fly strains were used in these experiments: *yolk-Gal4* [15]; w[*]; *UAS-Chmp1*; *D/TM3Ser* [16]; w[*]; *P{w[+mC]=UAS-GFP.S65T}Myo31DF[T2]* (referred to as *UAS-GFP*; Bloomington #1521); *y[1] v[1]*; *P{y[+t7.7] v[+t1.8]=TRiP.HM05117}attP2* (referred to as *UAS-Chmp1-RNAi*; Bloomington #28906); *y[1] v[1]*; *P{y[+t7.7]=CaryP}attP2* (*UAS-Chmp1-RNAi* genetic background control; Bloomington #36303). Flies were grown on a 12 h:12 h light:dark cycle at 25 °C on cornmeal-sugar-yeast medium as described previously [17].

### 2.2. Protein, Triglyceride, and DNA Measurements

Approximately 1-week-old female flies were used throughout this study. Two whole flies or three dissected abdomen cuticles with fat bodies attached were homogenized in lysis buffer (140 mM NaCl, 50 mM Tris-HCl, pH 7.4, 0.1% Triton-X, and 1X protease inhibitor (ThermoFisher, Waltham, MA, USA)). Triglyceride, protein, and DNA were measured using the Infinity Triglyceride Reagent (ThermoFisher, Waltham, MA, USA), Pierce BCA Protein Assay kit (ThermoFisher, Waltham, MA, USA) and Quant-iT DNA Assay kit (Invitrogen, Waltham, MA, USA), respectively, accordingly to manufacturer’s instructions and as described previously [17,18].

### 2.3. CAFÉ Assay

Food consumption was measured using the capillary feeding (CAFÉ) assay [19]. Briefly, three 1-week-old female flies were housed in a vial with 1% agar as a water source. Flies were fed 5% sucrose in a Drummond 5 μL capillary tube (ThermoFisher, Waltham, MA, USA as their sole food source and the amount of sucrose consumed was measured after 24 h. Vials without flies were used to account for any sucrose evaporation.

### 2.4. Gene Expression Analysis

RNA isolation, DNase treatment and reverse transcription were performed on abdomen cuticles with fat bodies attached dissected from 15 one-week-old female flies as previously described [17]. qPCR reactions were generated using 1 μL of cDNA, 200 nM primers, 1 × Perfecta SYBR Green (Quanta Biosciences, Gaithersburg, MD, USA in a 25 μL reaction. qPCR was performed on a StepOnePlus instrument using the following cycling conditions: initial denaturation at 95 °C for 3 min, 40 cycles of 30 s at 95 °C, 60 s at 60 °C and 30 s at 72 °C followed by a melt curve. Expression of all genes were normalized to *rp49*. Primer sequences used were: *Chmp1* (For 5′-GGCCTCGAACTCAACATGGA-3′ and Rev 5′-CAGAAATTCAACGGGCAGGG-3′), *FASN1* (For 5′-CTGGCTGAGCAAGATTGTGTG-3′ and Rev 5′-TCGCACAACCAGAGCGTAGTA), *ATPCL* (For 5′-CACGACAGATTGGTCCAAGCTC-3′ and Rev 5′-CTTGCTCTTCACGTCGGCTAAC-3′), *bmm* (For 5′-ACGTGATCATCTCGGAGTTTG-3′ and Rev 5′-ATGGTGTTCTCGTCCAGAATG-3′), *whd* (For 5′-GCCAATGTGATTTCCCTGCTTC-3′ and Rev 5′-CTTTGCCCTTCAGGATTTCCTC-3′) and *rp49* (For 5′-GACGCTTCAAGGGACAGTATCTG-3′ and Rev 5′-AAACGCGGTTCTGCATGAG-3′).

### 2.5. Statistics

The results were expressed as mean ± standard error (SE) and average values were compared between Chmp1-RNAi or Chmp1 overexpressing flies and the appropriate controls using a two-tailed Student’s *t*-test as calculated in Microsoft Excel. *p* < 0.05 was used as the cut off for statistical significance.

## 3. Results

### 3.1. Chmp1 Acts in the Drosophila Fat Body to Regulate Triglyceride Storage

**Figure 1:**
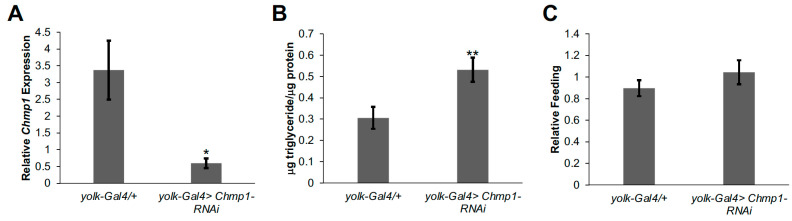
Decreasing *Chmp1* expression in the *Drosophila* fat body results in triglyceride accumulation. (**A**) Fat body *Chmp1* expression, (**B**) μg triglyceride/μg protein levels and (**C**) food consumption over 24 h were measured in 1-week-old *yolk-Gal4>Chmp1-RNAi* females and compared to *yolk-Gal4/+* controls. Bars indicate mean ± standard error. *, *p* < 0.05, **, *p* < 0.01 by Student’s *t* test.

To assess the role of fat body *Chmp1* in regulating lipid storage in *Drosophila*, we decreased *Chmp1* levels by inducing RNAi towards *Chmp1* specifically in the fat body using the previously described *yolk-Gal4* driver [15] and *UAS-Chmp1-RNAi* fly line (Figure 1A; [16]). The *yolk-Gal4* driver crossed to the *UAS-Chmp1-RNAi* genetic background stock was used as a control. Triglycerides were measured in *Chmp1-RNAi* flies and controls, and we observed an increase in triglyceride storage in fat-body-specific *Chmp1* knockdown flies (Figure 1B). This accumulation of triglycerides was not due to increased feeding as food consumption was not altered in *Chmp1-RNAi* flies (Figure 1C).

**Figure 2:**
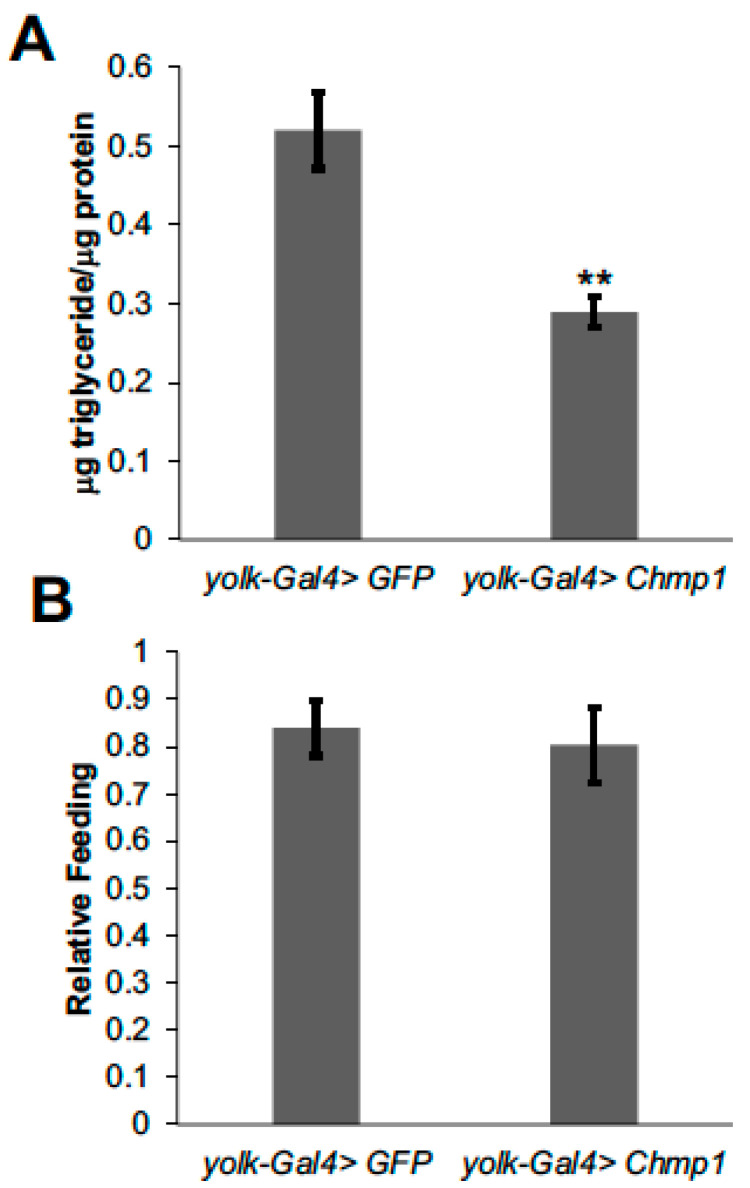
Overexpressing *Chmp1* in the *Drosophila* fat body blunts triglyceride storage. (**A**) μg triglyceride/μg protein levels and (**B**) food consumption over 24 h were measured in 1-week-old *yolk-Gal4>Chmp1* females and compared to *yolk-Gal4>GFP* controls. Bars indicate mean ± standard error. ** *p* < 0.01 by Student’s *t* test.

To further investigate the role of *Chmp1* in regulating fat body lipid storage, we overexpressed *Chmp1* in the female fat body using *yolk-Gal4* and the previously described *UAS-Chmp1* fly line [16] and compared them to control flies expressing green fluorescent protein (GFP). Conversely to the *Chmp1-RNAi* flies, overexpressing *Chmp1* blunts triglyceride storage (Figure 2A), and this is not due to decreased food consumption (Figure 2B). Together, the *Chmp1-RNAi* and *Chmp1* overexpression data indicate a role of *Chmp1* in limiting lipid levels in the *Drosophila* fat body.

### 3.2. Chmp1 Does Not Alter the Expression of Lipid Metabolic Enzyme Genes in the Drosophila Fat Body

**Figure 3:**
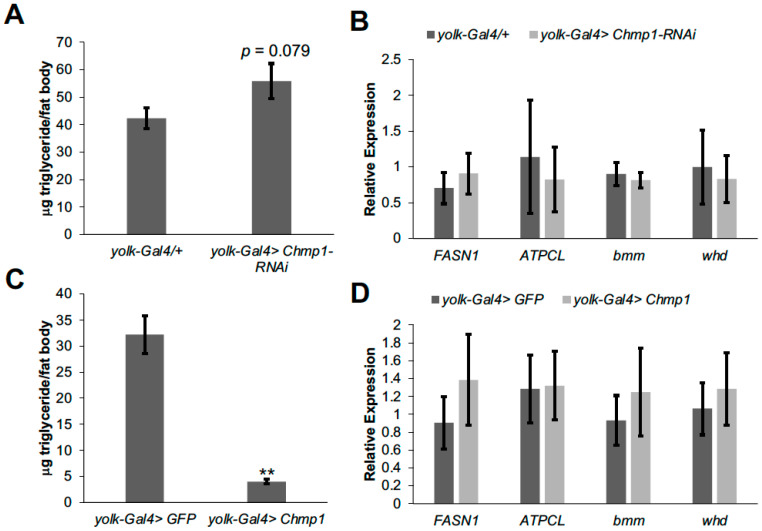
Fat body *Chmp1* regulates triglyceride storage but not by altering lipid metabolic enzyme gene expression. μg triglyceride/μg protein levels were measured in cuticles with fat bodies attached dissected from abdomens of 1-week-old female (**A**) *yolk-Gal4>Chmp1-RNAi* or (**C**) *yolk-Gal4>Chmp1* flies and compared to those dissected from (**A**) *yolk-Gal4/+* or (**C**) *yolk-Gal4>GFP* controls. qPCR was performed for *FASN1*, *ATPCL*, *bmm*, and *whd* in cuticles with fat bodies attached dissected from abdomens of 1-week-old female (**B**) *yolk-Gal4>Chmp1-RNAi* or (**D**) *yolk-Gal4>Chmp1* flies and compared to those dissected from (**B**) *yolk-Gal4/+* or (**D**) *yolk-Gal4>GFP* controls. Expression of all genes was normalized to *rp49*. Bars indicate mean ± standard error. **, *p* < 0.01 by Student’s *t* test.

To determine whether the defects in lipid storage in *Chmp1-RNAi* and *Chmp1* overexpressing flies were autonomous to the fat body, we dissected abdomens with fat bodies attached from these flies and measured triglyceride levels. There was a trend for an increase in triglycerides in fat bodies dissected from *Chmp1-RNAi* flies (Figure 3A) and there was a strong decrease in triglycerides in fat bodies dissected from *Chmp1* overexpressing flies (Figure 3C), consistent with an autonomous role of *Chmp1* in the fly fat body and the triglyceride storage results from whole flies. To further investigate the role of *Chmp1* in regulating fat body lipid storage, we wanted to test whether the triglyceride storage defects in *Chmp1-RNAi* and *Chmp1* overexpressing flies were due to changes in the expression of lipid metabolic enzyme genes. To address this question, we dissected abdomen cuticles with fat bodies attached, isolated RNA, and performed qPCR for two genes important for lipid synthesis (*FASN1* and *ATPCL*) and two genes important for lipid breakdown (the lipase *bmm* and *whd*, the fly homolog of the β-oxidation gene carnitine palmitoyltransferase (CPT1)). Interestingly, the expression of each of these genes was normal in *Chmp1-RNAi* fat bodies (Figure 3B) and *Chmp1* overexpressing fat bodies (Figure 3D) suggesting that altered expression of these lipid metabolic enzyme genes is not responsible for the triglyceride storage phenotypes observed in these flies.

### 3.3. Chmp1 Regulates Fat Body Cell Number and Size and the Amount of Stored Fat Body Triglyceride

**Figure 4:**
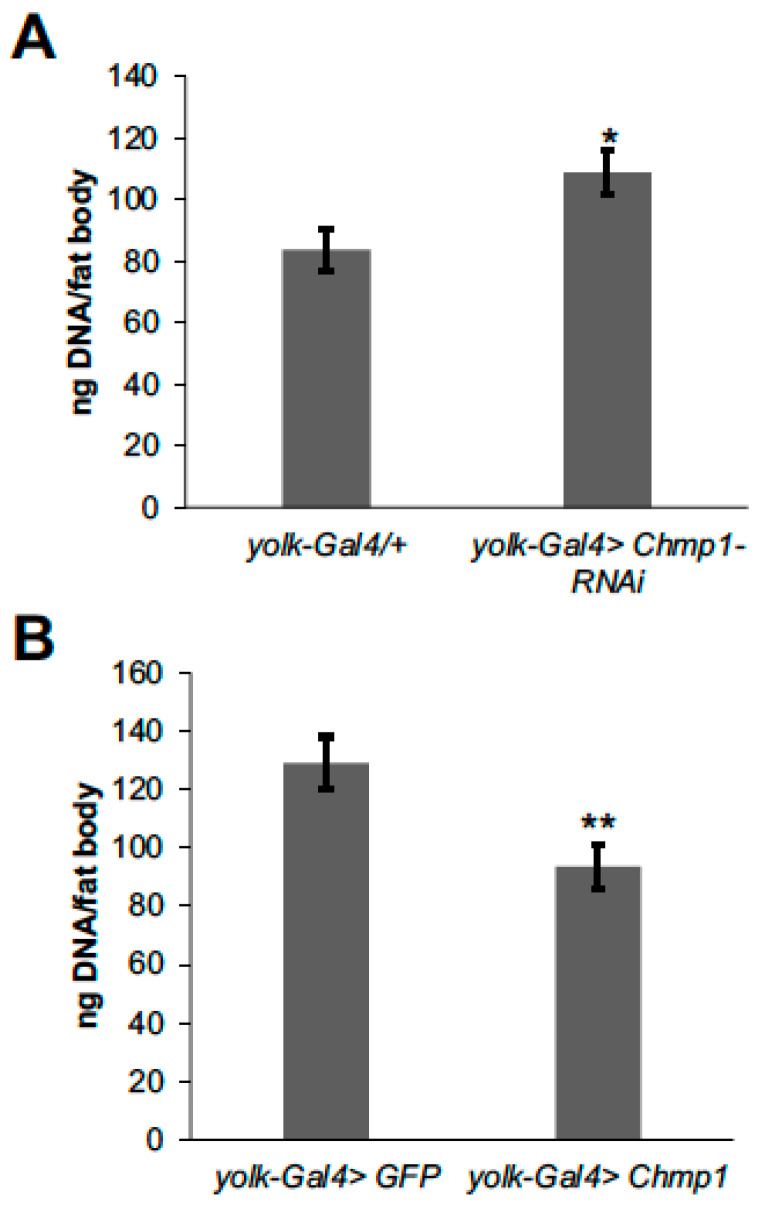
Fat body *Chmp1* regulates fat cell number. Fat body DNA content was measured in 1-week-old female (**A**) *yolk-Gal4>Chmp1-RNAi* flies and *yolk-Gal4/+* controls and (**B**) *yolk-Gal4>Chmp1* flies and *yolk-Gal4>GFP* controls. Bars indicate mean ± standard error. *, *p* < 0.05, **, *p* < 0.01 by Student’s *t* test.

Another potential cause of the alterations in fat body lipid storage in flies with altered *Chmp1* levels could be changes in the number or size, fat body cell triglyceride storage or a combination of these. To test whether *Chmp1* regulates the number of fat body cells, fat body DNA levels were measured as a proxy for fat cell number as previously described [18]. Fat body DNA was increased in *Chmp1-RNAi* flies (Figure 4A) suggesting that the triglyceride accumulation observed in these flies could be due to more fat body cells being produced. Conversely, *Chmp1* overexpressing flies had decreased total DNA content (Figure 4B) indicating that having fewer fat body cells contributes to the lean phenotype observed in these flies.

**Figure 5:**
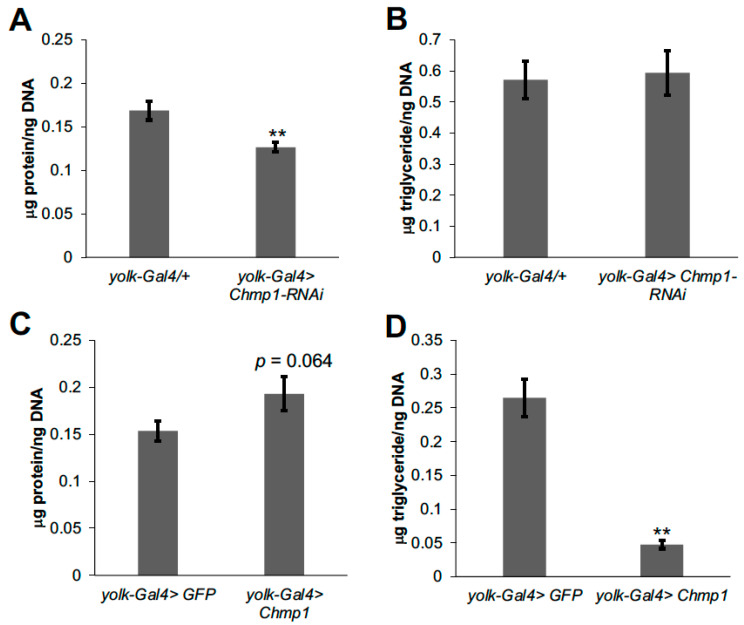
Fat body *Chmp1* regulates fat cell size. Total fat body protein normalized to DNA content was measured in 1-week-old female (**A**) *yolk-Gal4>Chmp1-RNAi* or (**C**) *yolk-Gal4>Chmp1* flies and compared to (**A**) *yolk-Gal4/+* or (**C**) *yolk-Gal4>GFP* controls. Fat body triglycerides normalized by total DNA were measured in 1-week-old female (**B**) *yolk-Gal4>Chmp1-RNAi* or (**D**) *yolk-Gal4>Chmp1* flies and compared to (**B**) *yolk-Gal4/+* or (**D**) *yolk-Gal4>GFP* controls. Bars indicate mean ± standard error. **, *p* < 0.01 by Student’s *t* test.

To determine whether *Chmp1* can regulate fat body cell size, total protein was normalized by DNA content which has been used previously as an indication of cell size [20]. Decreasing *Chmp1* in the fat body resulted in a lower protein/DNA ratio than controls (Figure 5A) and *Chmp1* overexpressing fat body cells were trending towards an increased protein/DNA ratio (Figure 5C) suggesting that *Chmp1* also regulates fat body cell size. Moreover, to determine whether fat body cell lipid storage was altered in *Chmp1-RNAi* and *Chmp1* overexpressing flies, fat body triglycerides were normalized by fat body DNA. Interestingly, while *Chmp1-RNAi* flies had no difference in fat body triglyceride normalized by DNA (Figure 5B), *Chmp1* overexpressing flies had a large decrease in triglyceride/DNA (Figure 5D). These data suggest that *Chmp1* is not necessary to control fat body cell triglyceride storage but sufficient to regulate fat body cellular triglycerides. Together, these data display a function of the ESCRT-III protein, *Chmp1*, in the regulation of triglyceride storage and fat cell number and size in the *Drosophila* fat body.

## 4. Discussion

In this study, we have uncovered a novel role for the ESCRT-III protein, *Chmp1*, in controlling lipid storage in the *Drosophila* fat body. Our data indicate that *Chmp1* functions to limit triglyceride levels as fat-body-specific *Chmp1* overexpression decreases triglyceride levels and *Chmp1-RNAi* increases it. These results are consistent with experiments performed in the yeast *Saccharomyces cerevisiae* showing that mutants in ESCRT complex genes result in less lipid droplet consumption and increased triglyceride storage [12]. In addition, experiments performed in cultured *Drosophila* cells revealed that knocking down *COPI* complex genes with RNAi results in larger lipid droplets [5,6], a phenotype similar to the increased triglycerides observed in *Chmp1-RNAi* fat bodies, suggesting a broader role for vesicular trafficking proteins in regulating lipid homeostasis. Arf79d, a member of the Arf1 family of monomeric G proteins that function in the endomembrane system, was also shown to localize to the lipid droplet surface and alter rates of lipolysis, suggesting a role for vesicular trafficking proteins in regulating lipid breakdown [6]. *Chmp1* has been shown to localize to endosomes in the developing fly wing [16], but whether *Chmp1* is found on the surface of lipid droplets and plays a role in the budding of lipid-laden vesicles during lipolysis in the *Drosophila* fat body is not known. Additional experiments designed to visualize whether *Chmp1* co-localizes with lipid droplets in *Drosophila* fat body cells and whether lipolysis is altered in *Chmp1-RNAi* or *Chmp1* overexpressing fat bodies would help address this question.

We have also shown that *Chmp1* controls fat body lipid storage by regulating the number and size of fat body cells. *Chmp1-RNAi* results in more smaller fat body cells suggesting that loss of *Chmp1* results in increased cell proliferation or decreased apoptosis. Decreasing the expression of the ESCRT-III protein gene *Vps4* in fly imaginal discs results in larval overgrowth, despite concomitant increases in apoptosis [21]. Since *Chmp1* binds to Vps4 in the ESCRT-III complex [13,14], perhaps *Chmp1* loss in the *Drosophila* fat body might also promote cell proliferation or growth to regulate fat body cell number and size. Measuring fat body cell proliferation and apoptosis in *Chmp1-RNAi* and *Chmp1* overexpressing flies will test this hypothesis.

*Chmp1* is also able to decrease the amount of fat stored per cell (as measured by fat body triglycerides normalized by DNA content) when it is overexpressed. This is consistent with a potential role for *Chmp1* in specifically promoting lipolysis as speculated above or lipid breakdown more generally, but independently of changes in the expression of important lipid breakdown genes like *bmm* or *whd* as their expression in unchanged in *Chmp1*-overexpressing flies. Interestingly, *Chmp1-RNAi* fat bodies have normal triglyceride storage when normalized to DNA, suggesting that *Chmp1* may not be limiting to regulate the amount of fat stored per cell under physiological conditions. Since *Chmp1* only alters the amount of triglyceride stored in each fat cell when it is overexpressed, *Chmp1* may only be sufficient to regulate fat cell lipid storage. This is consistent with experiments manipulating *Chmp1* in the developing fly wing where *Chmp1* overexpression revealed additional phenotypes not observed in *Chmp1-RNAi* wings [16]. It is possible that ESCRT complex activity is altered to different degrees in *Chmp1-RNAi* and *Chmp1* overexpressing fat bodies causing differential regulation of the amount of fat stored per cell. It is also possible that *Chmp1* overexpression might uncover additional signaling pathways regulated by *Chmp1*. Experiments designed to measure ESCRT complex activity in *Chmp1-RNAi* and *Chmp1* overexpressing fat bodies would help differentiate among some of these possibilities.

One of the major functions of ESCRT proteins is to identify monoubiquitinated receptors for signaling pathways, remove them from the plasma membrane and traffic them to endosomes, ultimately leading to degradation in the lysosome [9,10]. Many ESCRT proteins in *Drosophila* have been shown to recognize monoubiquitinated signaling receptors such as epidermal growth factor receptor (Egfr) and Notch and target them to the multivesicular body in endosomes [22]. In ESCRT mutants in *Drosophila*, these receptors remain on the plasma membrane and their signaling pathways are more active compared to wildtype controls [16,22]. Consistent with this signaling receptor accumulation, decreasing *Chmp1* levels in the larval fat body results in the production of small lysosomes and decreased autophagy indicating less protein degradation [23]. However, whether Egfr or Notch signaling is altered in *Chmp1-RNAi* or *Chmp1* overexpressing fat bodies and whether this signaling may contribute to the triglyceride storage phenotypes observed here is not known. Previous studies have shown that activating the insulin receptor in the *Drosophila* fat body results in an increase in the number of fat cells and an increase in the amount of triglyceride stored in each fat cell [18]. Since we have shown here that *Chmp1* can regulate both fat cell number and fat cell triglyceride storage, it is possible that *Chmp1* may recognize a monoubiquitinated insulin receptor and decrease insulin signaling in fat body cells, altering fat cell production or fat cell lipid storage. Performing experiments measuring whether insulin signaling activity is increased in *Chmp1-RNAi* fat bodies and decreased in *Chmp1* overexpressing fat bodies would help support this hypothesis.

Previous work from our lab has elucidated a role for *Chmp1* in regulating the transport of polyamines in *Drosophila* using a leg imaginal disc system [24]. Polyamines are small nitrogen-containing molecules that regulate many biological processes including cell differentiation and proliferation [25,26]. In addition, polyamines have also been implicated in regulating lipid metabolism in the *Drosophila* fat body as well as in mammalian adipocytes [27,28]. This raises the possibility that perhaps *Chmp1* may also be regulating polyamine transport in the fly fat body and this may contribute to the lipid metabolic phenotypes observed here.

In summary, we have described a novel metabolic role for the ESCRT-III protein, *Chmp1*, in the *Drosophila* fat body. *Chmp1* is highly conserved from yeast to humans [10], so the results shown here could be used to increase our understanding of lipid metabolism in humans. In addition, one of the human *Chmp1* homologs, Chmp1A, has been shown to be important in several cancers [29,30] potentially broadening the interest of this work. Together, these results support the expanding roles of the endomembrane system and vesicular trafficking proteins in the regulation of overall lipid homeostasis.

## Data Availability

All of the data presented here are contained within the article.
